# Gelatin Methacryloyl Hydrogels for the Localized Delivery of Cefazolin

**DOI:** 10.3390/polym13223960

**Published:** 2021-11-16

**Authors:** Margaux Vigata, Cathal D. O’Connell, Silvia Cometta, Dietmar W. Hutmacher, Christoph Meinert, Nathalie Bock

**Affiliations:** 1 Faculty of Engineering, School of Mechanical, Medical and Process Engineering, Queensland University of Technology, Brisbane, QLD 4000, Australia; margaux.vigata@yahoo.fr (M.V.); silviacatalina.comettaconde@hdr.qut.edu.au (S.C.); dietmar.hutmacher@qut.edu.au (D.W.H.); 2 Aikenhead Centre for Medical Discovery (ACMD), St Vincent’s Hospital Melbourne, Fitzroy, VIC 3065, Australia; 3 Discipline of Electrical and Biomedical Engineering, School of Engineering, RMIT University, Melbourne, VIC 3000, Australia; 4 Australian Research Council Training Centre for Multiscale 3D Imaging, Modelling and Manufacturing (M3D Innovation), Queensland University of Technology, Kelvin Grove, QLD 4059, Australia; 5 Faculty of Health, School of Biomedical Sciences, Queensland University of Technology, Brisbane, QLD 4000, Australia; 6 Australian Research Council Industrial Transformation Training Centre in Additive Biomanufacturing, Queensland University of Technology, Brisbane, QLD 4059, Australia; 7 Australian Research Council Training Centre for Cell and Tissue Engineering Technologies, Queensland University of Technology, Brisbane, QLD 4059, Australia; 8 Herston Biofabrication Institute, Metro North Hospital and Health Services, Herston, QLD 4006, Australia; 9 Translational Research Institute, Woolloongabba, QLD 4102, Australia

**Keywords:** gelatin methacryloyl, cefazolin, localized antibiotic therapy, drug delivery, surgery site infection

## Abstract

The tuneability of hydrogels renders them promising candidates for local drug delivery to prevent and treat local surgical site infection (SSI) while avoiding the systemic side-effects of intravenous antibiotic injections. Here, we present a newly developed gelatin methacryloyl (GelMA)-based hydrogel drug delivery system (GelMA-DDS) to locally deliver the broad-spectrum antibiotic cefazolin for SSI prophylaxis and treatment. Antibiotic doses from 3 µg to 90 µg were loaded in photocrosslinked GelMA hydrogel discs with 5 to 15% *w/v* polymer concentration and drug encapsulation efficiencies, mechanical properties, crosslinking and release kinetics, as well as bacterial growth inhibition were assessed. Our results demonstrate that all GelMA groups supported excellent drug encapsulation efficiencies of up to 99%. Mechanical properties of the GelMA-DDS were highly tuneable and unaffected by the loading of small to medium doses of cefazolin. The diffusive and the proteolytic in vitro drug delivery of all investigated cefazolin doses was characterized by a burst release, and the delivered cefazolin amount was directly proportional to the encapsulated dose. Accelerated enzymatic degradation of the GelMA-DDS followed zero-order kinetics and was dependent on both the cefazolin dose and GelMA concentration (3–13 h). Finally, we demonstrate that cefazolin delivered from GelMA induced a dose-dependent antibacterial efficacy against *S. aureus*, in both a broth and a diffusive assay. The cefazolin-loaded GelMA-DDS presented here provides a highly tuneable and easy-to-use local delivery system for the prophylaxis and treatment of SSI.

## 1. Introduction

Medical procedures present an inherent risk of surgical site infection (SSI) [1]. Hospital-acquired infections, also known as nosocomial infections, are the most frequent SSI reported. SSI represents 15–20% of all nosocomial infections and, hence, have a significant economic and human impact [2,3]. The most common pathogens implicated in SSI are *S. aureus*, *S. epidermidis*, and Coagulase-negative *Staphylococci*, but an increasing number of SSI reported are attributed to methicillin-resistant *S. aureus* (MRSA) [1,3,4].

Cefazolin is amongst the most commonly used antibiotic agents to combat and prevent SSI [3,5,6,7]. It is a bactericidal first-generation beta-lactam antibiotic that binds to penicillin-binding proteins, which are the catalyzer for peptidoglycan synthesis, and thus disrupts the bacteria wall formation, causing the bacteria to lyze [8]. Cefazolin is often used perioperatively due to its effectiveness against a wide range of gram positive (*Staphylococci* and *Streptococci*) and gram-negative bacteria (*Escherichia coli* or *Proteus mirabilis*) [3,5,6]. Despite its common usage, systemic administration of cefazolin can lead to severe side effects ranging from allergic reactions to encephalopathy or epilepsy [9]. Additionally, a growing concern is the potential for acquired antibiotic resistance resulting from systemic administration of cefazolin [10,11,12,13].

Controlled local delivery of drugs has been proposed as an approach to overcome the risks associated with antibiotic overuse, since drugs delivered locally require significantly lower doses compared to systemic approaches [14]. Local drug delivery systems (DDS) can effectively control drug bioavailability to target cells and tissue over time and space to leverage beneficial outcomes of therapeutics enhancing efficacy and reducing toxicity and required dosage [15]. Various DDS classes have been explored in scientific literature, including membranes [16], nanoparticles [17], liposomes [18], and hydrogels [15]. Hydrogels are a particularly interesting material class for DDS and have been extensively used in many branches of medicine and tissue engineering. Hydrogels are composed of crosslinked polymer networks that hold large amounts of water, often ranging from 70 to 99% of wet weight. This high-water content provides similarities to native tissue and excellent biocompatibility, as well as the ability to easily retain hydrophilic drugs. Furthermore, because of their formation in aqueous conditions, risks associated with drug aggregation and denaturation are minimized [15]. Hydrogels provide excellent tuneability of their physical properties that enable precise matching of soft tissue properties in the human body [19]. On a nanometre scale, crosslinked hydrogel networks provide tuneable porosity, referred to as the mesh size, which governs and controls drug diffusion within and from hydrogel-based DDS. Therefore, the modularity, intrinsic properties, and tuneability of hydrogels enable precise tailoring of drug release in time and space, rendering this class of materials promising for clinical local drug delivery applications [15].

In this work, we developed a hydrogel DDS based on gelatin methacryloyl (GelMA) for the local release of cefazolin. To retain simplicity and clinical relevance, the antibiotic was loaded with the GelMA hydrogel precursor solution before photocrosslinking. GelMA is a semi-synthetic hydrogel that retains the advantages of polymers of natural origin, i.e., biocompatibility, low immunogenicity, and biodegradability, owing to its natural metalloprotease-cleavable motifs. The functionalization of gelatin by the addition of methacryloyl groups enables enhanced control over the physicochemical properties of the hydrogel [20]. Additionally, GelMA is a well-described biomaterial to which physical properties can be tailored based on crosslinking conditions [21]. The stiffness of the hydrogel is directly proportional to the mesh size of the hydrogel matrix, which, in turn, is used to tune the release of therapeutic molecules [22]. Li et al. described the release mechanisms of drugs from hydrogel polymers: diffusion through the matrix, swelling, or degradation. Drugs smaller than the hydrogel mesh size will follow a fast diffusion, while drugs of similar or larger size will be slowed down and/or retained. In the latter case, the therapeutic molecule release can follow other mechanisms involving hydrogel deformation, swelling, or degradation [15].

We hypothesized that different GelMA concentrations would allow the ability to tailor physical properties such as matrix stiffness, porosity, water content, permeability, and diffusive properties, which have previously been shown to have an effect on the antibiotic release kinetics in other hydrogel systems [22,23]. We first evaluated the in vitro release of cefazolin in phosphate-buffered saline (PBS) and following collagenase degradation. The release kinetics characterization was completed by assessing the hydrogels’ swelling properties and related mesh size. Then, we characterized the mechanical properties of the GelMA-DDS loaded with several cefazolin doses, as well as the impact of cefazolin on the gelation time and polymerization kinetics. Finally, we studied the in vitro antibacterial efficacy of the cefazolin-loaded GelMA-DDS in a zone of inhibition assay and a broth assay of the bacterium *S. aureus*.

## 2. Material and Methods

### 2.1. Drug Encapsulation and GelMA Crosslinking

Gelatin methacryloyl (GelMA; porcine skin, type A, ~80% degree of functionalization) was provided by Gelomics (Gelomics, Brisbane, Australia). GelMA-drug delivery system (DDS) samples for in vitro assays were manufactured using a mold casting technique. Custom polytetrafluoroethylene (PTFE) molds were used to make disc-shaped samples measuring 5 mm diameter and 1.8 mm height and ~35 µL of volume. Hydrogel discs based on 5%, 10%, or 15% GelMA containing 0 µg, 3 µg, 15 µg, 30 µg, or 90 µg cefazolin (Sigma-Aldrich, St Louis, MO, USA) (Table 1), respectively, were prepared by ultraviolet (UV) crosslinking at 365 nm for 30 min (intensity of ~2.6 mW/cm^2^ in a CL-1000 crosslinker; UVP, Upland, CA, USA) in the presence of and 0.05 % *w/v* Irgacure 2959 (1-[4-(2-hydroxyethoxy)-phenyl]-2-hydroxy-2-methyl-1-propanone, BASF, Ludwigshafen, RLP, Germany). Hydrogel samples were stored at 4 °C until use.

### 2.2. Release Study

Drug release studies were carried out in PBS at pH 7.4, 37 °C, and under 75 rpm agitation in an orbital incubator. Each hydrogel sample (n = 6) was immersed in 0.5 mL of PBS in low protein binding tubes (Eppendorf Protein LoBind Tubes, Hamburg, Germany), and the entire volume was sampled and refreshed at the different timepoints.

#### 2.2.1. Drug Detection

Cefazolin quantification was performed using ultra-high-performance liquid chromatography with tandem mass spectrometry (UHPLC-MS-MS) using a Nexera X2 UHPLC system coupled to a triple quadrupole mass spectrometer Shimadzu LCMS-8050 (Shimadzu, Kyoto, Japan). All samples were filtered using 0.2 µm pore size syringe filters. The separation flow rate was of 0.3 mL/min using a Kinetex^®^ 2.6 μm EVO C18 100 Å LC column 100 × 2.1 mm (Phenomenex, Tokyo, Japan). The mobile phase A was water with 0.1% formic acid, while the mobile phase B consisted of 0.1% formic acid in acetonitrile. Samples were placed in the autosampler, in which temperature was set at 5 °C, and the injection volume was 1 µL. Cefazolin ionization was executed by a positive-ion electrospray with a nebulizing gas flow of 3 L/min, the heating gas flow of 10 L/min, drying gas flow of 10 L/min, interface temperature of 300 °C, desolvation line (DL) temperature of 250 °C, and heat block temperature of 400 °C.

#### 2.2.2. Encapsulation Efficiency

Drug-loaded hydrogel samples (n = 6) were degraded overnight at 37 °C and 300 rpm on a Thermomixer Compact (Eppendorf, Hamburg, Germany) in 0.5 mL Hank’s Balanced Salt Solution (Gibco™ HBSS, calcium, magnesium, no phenol red, Thermo Fischer Scientific, Waltham, MA, USA) containing 28 units/mL collagenase II (Gibco™ Collagenase, Type II powder, Thermo Fischer Scientific, Waltham, MA, USA). Cefazolin drug quantification was performed with HPLC-MS-MS as outlined above. Supplementary Figure S1 shows an offset in the cefazolin detection used to create standard curves between the drug detected in a PBS solution versus a collagenase-HBSS solution. The detection of cefazolin in collagenase and HBSS solution was, on average, 13.37% lower. Consequently, the encapsulation efficiencies measured were corrected by a factor of 1.1337.

#### 2.2.3. Degradation Characterization

To simulate in vivo environments and investigate the release of cefazolin in response to proteolytic degradation, accelerated enzymatic degradation of the GelMA hydrogels was performed in vitro using collagenase. Hydrogel samples (n = 4) were digested at 37 °C, at 300 rpm, in 0.5 mL of 28 units/mL collagenase (Gibco™ Collagenase, Type II powder, Thermo Fischer Scientific, Waltham, MA, USA) in Hank’s Balanced Salt Solution (Gibco™ HBSS, calcium, magnesium, no phenol red, Thermo Fischer Scientific, Waltham, MA, USA). Every two hours, the hydrogels were weighed, and the collagenase solution was refreshed. The degradation rate was calculated using the ratio of the digested mass over the original mass.

#### 2.2.4. Release via Degradation

Samples (n = 3) were digested in 0.5 mL of 28 units/mL collagenase II (Gibco™ Collagenase, Type II powder, Thermo Fischer Scientific, Waltham, MA, USA) at 37 °C and 300 rpm on a thermomixer compact (Eppendorf, Hamburg, Germany). Every two hours, the digestion media was sampled for drug detection and refreshed. Drug detection was carried out using HPLC-MS-MS.

#### 2.2.5. Equilibrium Mass Swelling Ratio and Mesh Size Calculation

##### Equilibrium Mass Swelling Ratio

For this study, two control groups were added: 5% GelMA and 15% GelMA. Samples (n = 5–7) were weighed after seven-day swelling to equilibrium in PBS at 37 °C, and then again after freeze-drying. The equilibrium mass swelling ratio (*Q_m_*) was calculated according to the following equation:

(1) $$Q_{m}=\frac{\left(m_{wet}-m_{lyophilized}\right)}{m_{lyophilized}}\;$$

where $m_{Wet}$ is the wet mass after swelling and $m_{lyophilized}$ is the mass after lyophilization of the hydrogel samples.

#### 2.2.6. Mesh Size Calculation

The mesh size ξ of the GelMA hydrogels was determined using the mass swelling ratio, and the inherent characteristics such as the molecular weight between crosslinks, the molecular weight of the repeating unit, the bond length, and the equilibrium polymer volume fraction ($v_{2s}$), of gelatin and GelMA hydrogels [24,25] in Equation (2).

Mesh size was calculated using the molecular weight of the repeating unit $M_{r}$ (averaged molecular weight of the amino acid composition, $M_{r}=91.19\;g/\mathrm{mol}$) [26], the amino acid bond length $l=4.28\;\dot{A}$ [27], the Flory’s characteristic ratio $C_{n}$ for GelMA was taken as 8.8785 [26], and the molecular weight between crosslinks $M_{c}\;$ was calculated using Equation (5).

(2) $$\xi =v_{2s}{}^{-\frac{1}{3}}\times l\;{\left(2\;\frac{M_{c}}{M_{r}}C_{n}\right)}^{\frac{1}{2}}\;\;\;\;$$

The relaxed volumetric swelling $Q_{vr}$ and the equilibrium volumetric swelling $Q_{v}$ were obtained by converting the respective relaxed mass swelling ratio $Q_{mr}$ and the equilibrium mass swelling ratio $Q_{m}$ using the gelatin density as polymer density ($\rho_{p\;}=1.35\;g/{\mathrm{cm}}^{3}$) [28,29,30] and the PBS density as solvent density ($\rho_{s\;}=1.014\;g/{\mathrm{cm}}^{3}$) [31] in Equation (3). The relaxed mass swelling ratio $Q_{mr}$ and the equilibrium mass swelling ratio $Q_{m}$ were obtained with Equation (1) using the wet weight just after crosslinking and after reaching equilibrium swelling, respectively.

(3) $$Q_{v\left(r\right)}=1+\frac{\rho_{p}}{\rho_{s}}\left(Q_{m\left(r\right)}-1\right)\;\;\;\;$$

Then the relaxed polymer volume fraction $v_{2r}$ and the equilibrium polymer volume fraction $v_{2s}$ were calculated using Equation (4).

(4) $$v=\frac{1}{Q_{v}}\;\;$$

Finally, the molecular weight between crosslinks $M_{c}$ (g/mol) was estimated using the Flory-Rhener Equation (5) used for polymers crosslinked in solvents [24,32,33]. The polymer properties such as the specific volume of the polymer $\bar{v}$ taken as 0.7407 mL/g [26], the molar volume of the solvent $V_{1}$ was 18.01 mL/mol for water [26], the polymer-solvent interaction $X_{1}$, which is known as the Flory’s Chi parameter, was 0.497 [34], and the number average molecular weight before crosslinks $M_{n}$ (63,565.35 g/mol) [35].

(5) $$\frac{1}{M_{c}}=\frac{2}{M_{n}}-\frac{\frac{\bar{v}}{V_{1}}\;[\ln\left(1-v_{2s}\right)+v_{2s}+X_{1}v_{2s}{}^{2}]}{v_{2r}\left[{\left(\frac{v_{2s}}{v_{2r}}\right)}^{\frac{1}{3}}-\frac{1}{2}\left(\frac{v_{2s}}{v_{2r}}\right)\right]}\;\;\;$$

### 2.3. Mechanical and Physical Properties

#### 2.3.1. Effective Mass Swelling

Samples (n = 8) were weighed immediately after crosslinking and then again after overnight swelling in PBS at 37 °C. The mass difference was expressed as a percentage to assess the effective mass swelling of the hydrogel-DDS:

(6) $$Effective\;swelling\;\left(\%\right)=\frac{\left(m_{Wet}-m_{initial}\right)}{m_{initial}}\times 100\%$$

where $m_{Wet}$ is the wet mass after swelling and $m_{initial}$ is the mass just after crosslinking and before swelling.

#### 2.3.2. Mechanical Compression Test

Compressive moduli, failure strain, failure stress, and toughness of GelMA-DDS samples (n = 8) were investigated in unconfined compression tests using an Instron 5848 microtester with a 500 N load cell (Instron, Melbourne, VIC, Australia). Before testing, samples were immersed overnight in PBS at 37 °C to facilitate swelling to equilibrium. During the test, samples were also immersed in PBS at 37 °C, and a non-porous aluminum indenter was used to apply compression with a displacement rate of 0.01 mm/s on the hydrogel samples. The slope of the strain-stress curve, from 10% to 15% strain, was used to determine the Young’s compressive modulus [20]. The maxima of the stress-strain curve before the hydrogel sample cracking (sudden drop in the stress-strain curve) determined the failure stress and strain. The toughness was defined as the area under the stress-strain curve from 0% strain until failure. A previously published automated algorithm was used for mechanical testing data analysis [36].

#### 2.3.3. Photorheology

Samples (n = 5) of the hydrogel precursor solution containing 0.05% Irgacure with and without cefazolin were analyzed on a rheometer (Physica MCR 302 Rheometer, Anton Paar, Graz, Austria), using a cone-plate configuration (15 mm disk, 1º cone angle, and a 31 µm truncation at the apex), at room temperature, (23 °C). Measurements were performed under oscillatory conditions at 0.5% strain and a frequency of 10 rad/s. The samples were photocrosslinked in situ with the aid of a UV light source (Omnicure S1000, Alpha UV Systems, Pymble, Australia) placed under a quartz crystal sample holder. An intensity of 365 nm was calibrated at 3 mW/cm^2^ using a radiometer (Omnicure R2000, Alpha UV Systems, Pymble, Australia). For a more comprehensive study, two groups were added: 5% GelMA-90 µg of cefazolin and 15% GelMA 90 µg of cefazolin, as well as their respective control groups: 5% GelMA and 15% GelMA.

### 2.4. Bacterial Culture

#### 2.4.1. Maintenance

*Staphylococcus aureus* (*S. aureus*) ATCC29213 was used for the different antibacterial assays. Lysogeny Broth (LB) agar plates were used for the culture.

#### 2.4.2. Zone of Inhibition Assay

A diffusion assay was performed using Mueller-Hinton (MH) agar-containing Petri dishes. A 0.5 McFarland standard solution [37] was prepared by suspending *S. aureus* colonies in MH broth and obtaining an optical density at 600 nm (OD_600_) between 0.08 and 0.1. To each MH Petrie dish, 30 µL of the solution was added and spread homogeneously. GelMA without loaded cefazolin and antimicrobial susceptibility test discs containing 30 µg of cefazolin (Oxoid, Thermo Fischer Scientific, MA, USA) were used as negative and positive controls, respectively. Plates were incubated overnight at 37 °C, plates were imaged, and the zones of inhibition were measured using Fiji ImageJ software. The experiment was performed three times with n = 6 each time.

#### 2.4.3. Broth Inhibition Assay

The minimum inhibitory concentration (MIC) of cefazolin for *S. aureus* ATCC29213 after 24 h was between 1.3 and 11.2 μg/mL for broth inoculum between 5 × 10^5^ CFU/mL and 5 × 10^7^ CFU/mL [38]. Therefore, we used an inoculum broth in the same range (5 × 10^6^ CFU/mL). We modified the assay, which usually lasts 24 h, to study the antibacterial efficacy of the cefazolin-loaded GelMA-DDS over a longer time-period by refreshing the bacteria broth every day for four days. The expectation was that all cefazolin doses encapsulated in the GelMA-DDS would successfully inhibit the bacteria growth for at least the first day of culture.

A 0.5 McFarland standard solution [37] was prepared in MH broth (5 × 10^8^ CFU/mL), and diluted to obtain a broth inoculum at 5 × 10^6^ CFU/mL. GelMA-DDS samples, a positive control, i.e., antimicrobial susceptibility test disc containing 30 µg of cefazolin (Oxoid, Thermo Fisher Scientific, MA, USA) and a free dose of 30 µg of cefazolin, as well as negative control (GelMA without cefazolin), were placed individually in the wells of a 48-well plate. The assay was launched upon the addition of 0.5 mL of prepared bacteria inoculum solution per well. At each timepoint (day 1, day 2, day 3, and day 4), the turbidity was measured using OD_600_, and a new bacteria broth solution was prepared to replace the 0.5 mL of media in each well completely. The experiment was performed three times with n = 6 each time.

### 2.5. Statistical Analysis

Probabilities of *p* ≤ 0.05 were considered a significant difference. Statistical analysis was performed by analysis of variance (ANOVA). The significance of mean differences between groups was calculated using a general linear model (univariate analysis) using IBM SPSS Statistics 23 (IBM Corp., Armonk, NY, USA).

## 3. Results and Discussion

### 3.1. Drug Release

In the first part of the study, we evaluated the drug encapsulation efficiencies and characterised in vitro drug release in PBS to provide diffusive release kinetics in physiological conditions. Furthermore, the in vitro drug release via accelerated enzymatic degradation was evaluated because metalloproteases degrade GelMA hydrogels in the body [39]. The equilibrium swelling and the GelMA-DDS mesh size were determined to complete the drug release characterization because the mesh size is the primary physical parameter able to control drug diffusion [15]. The second part focused on the impact of drug loading on the mechanical and swelling properties of the GelMA-DDS because these physical properties are main drivers of drug diffusion [22]. To further investigate the effect of drug loading on the crosslinking reaction of the GelMA-DDS, in situ photorheology experiments were conducted. Finally, in the third and last section, we evaluated the antibacterial efficacy of the cefazolin-loaded GelMA-DDS in a zone of inhibition and a broth assay against the model bacteria *S. aureus*.

#### 3.1.1. Encapsulation Efficiency

**Figure 1:**
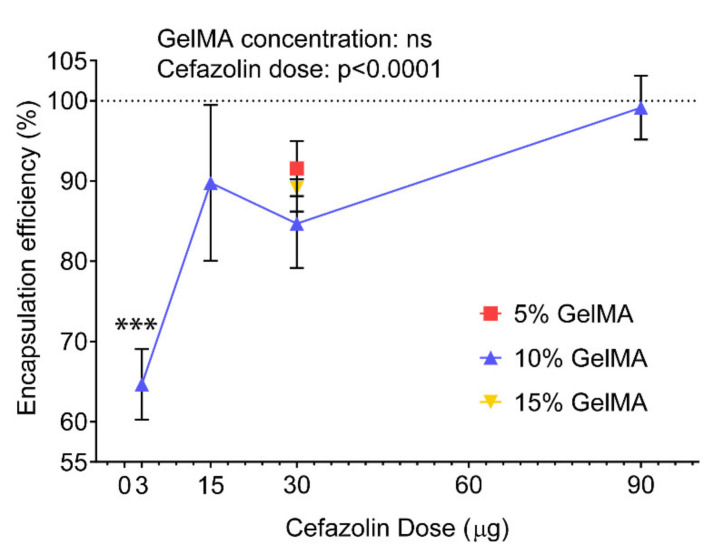
Encapsulation efficiencies of cefazolin in 5%, 10%, and 15% gelatin methacryloyl (GelMA) hydrogels prepared by blend-loading. Results are shown as mean ± standard deviation, n = 6. *** *p* < 0.001 compared to all other groups.

We first investigated the encapsulation efficiencies (EE) of cefazolin, defined as the percentage of cefazolin successfully encapsulated in our GelMA-DDS compared to the initial theoretical amount of drug loaded (Figure 1). In our study, EEs of cefazolin in the GelMA-DDS based on 10% GelMA were ≥84% for all drug doses, except for 3 µg cefazolin which averaged at 64.7%. The EE of 30 µg cefazolin was similar between 5%, 10% and 15% GelMA, suggesting that the macromolecule concentration did not influence this parameter.

The EEs obtained here were significantly higher compared to work previously published work by Shah et al., who obtained limited loading efficiency for cefazolin in poly(DL-lactic-co-glycolic acid) (PLGA) nanoparticles [40]. In their study, Shah et al. attributed the poor EE to a combination of charge incompatibility and polymer phase concentration between the negatively charged cefazolin and poly(DL-lactic-co-glycolic acid) (PLGA) microparticles, which are relatively hydrophobic [40]. The increased EEs obtained using GelMA as a drug carrier suggest that hydrogels present a more suitable environment for cefazolin encapsulation, a finding that is in accordance with other studies [41,42,43].

#### 3.1.2. Diffusive Release

Different doses of cefazolin (3µg, 15 µg, 30 µg, and 90 µg) were loaded in 10% GelMA hydrogels to evaluate the impact of the dose parameter on the diffusive drug release in PBS. To evaluate the effects of GelMA concentrations on antibiotic release, different concentrations of GelMA (5%, 10%, and 15%) were used to encapsulate the same dose of cefazolin (30 µg). Other studies have demonstrated that increasing the GelMA concentration decreases hydrogel mesh size [26,44], leading to higher stiffness [22] and lower permeability [22]. Notably, such tailoring of the GelMA crosslinking conditions was used to control the release of the anticancer drug doxorubicin, which is of similar molecular weight to cefazolin [45,46]. Different crosslinking times were applied to photo-cure the doxorubicin-loaded GelMA-DDS, thereby tailoring the GelMA-DDS mesh size and, ultimately, the doxorubicin release was a more sustained release with the stiffer GelMA-DDS groups [23].

**Figure 2:**
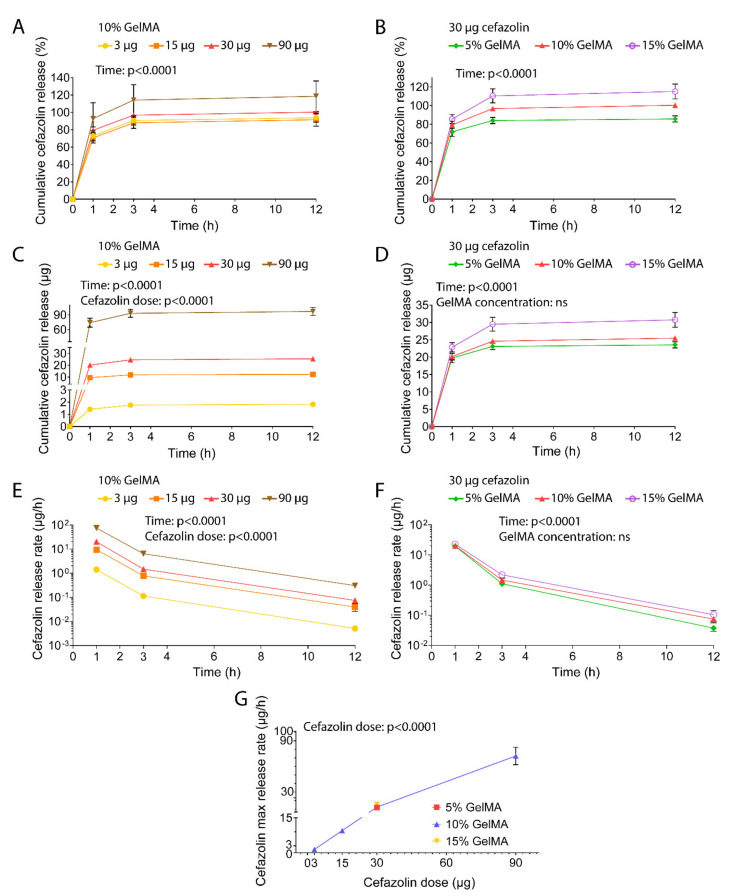
In vitro release of cefazolin in phosphate-buffered saline at pH 7.4, under 75 rpm agitation at 37 °C. (**A**) Cefazolin cumulative release for 3 µg, 15 µg, 30 µg, and 90 µg from 10% GelMA expressed as percentage of the encapsulation efficiency. (**B**) Cefazolin cumulative release for 30 µg from 5%, 10%, and 15% GelMA expressed as percentage of the encapsulation efficiency. (**C**) Cefazolin cumulative release for 3 µg, 15 µg, 30 µg, and 90 µg from 10% GelMA expressed in µg. (**D**) Cefazolin cumulative release for 30 µg from 5%, 10%, and 15% GelMA expressed in µg. (**E**) Cefazolin release rate per hour for 3 µg, 15 µg, 30 µg, and 90 µg from 10% GelMA expressed in µg/hour. (**F**) Cefazolin release rate per hour for 30 µg from 5%, 10%, and 15% GelMA expressed in µg/h. (**G**) Cefazolin maximum release rate at 1 h expressed in µg/h. Data are shown as mean ± standard deviation. The experiment was performed once with n = 6 technical replicates.

The release profiles of cefazolin at different doses and concentrations all displayed a burst release via diffusion (Figure 2A–D). Overall, around 70% to 92% of the drug cargo was released within one hour of incubation in PBS. In all cases, the total drug cargo was released within the first 12 h of the assay, suggesting that the cefazolin molecules diffuse out of the hydrogel matrix with ease. The GelMA concentration did not impact the release kinetics, whereas the cefazolin dose was a significant parameter that allowed us to tailor drug delivery (Figure 2C,D). The corresponding release rates (Figure 2E,F) displayed a maximum release rate between 0 and 1 h, which decreased following an exponential decay. Analysis of release rates confirmed burst release and its dependency on the cefazolin dose, but not on the GelMA concentration. The maximum cefazolin release rates increased from 1.42 µg/h to 74 µg/h in a dose-dependent manner, demonstrating that release could be tailored by amount of cefazolin loaded in the GelMA-DDS (Figure 2G and Supplementary Figure S2).

Contrary to Luo et al.’s study, our results did not show control over drug release kinetics when modifying the crosslinking conditions of the GelMA-DDS. This is likely explained by the nature of the drug used in this study, doxorubicin, a highly hydrophobic molecule [47], versus cefazolin that presents high hydrophilicity. It is cefazolin’s high water solubility that favors its rapid diffusion from the hydrogel into the aqueous release media [45]. Our results therefore suggest that not only the physical tailoring of the hydrogel-DDS should be considered but also the drug hydrophilicity, which dictates its stability and affinity for the hydrogel polymer and released media [48].

#### 3.1.3. Release via Enzymatic Degradation

**Figure 3:**
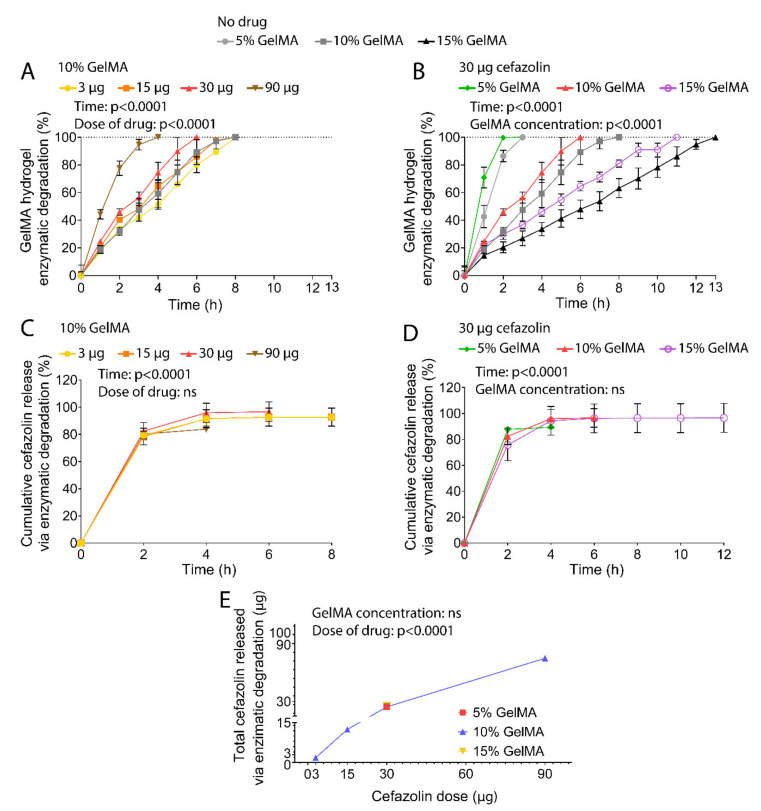
In vitro proteolytic release of cefazolin via collagenase II degradation of GelMA hydrogels at 28 units/mL under 300 rpm agitation at 37 °C. (**A**) Hydrogel degradation defined as mass loss normalized to initial mass for 5%, 10%, and 15% GelMA and 3 µg, 15 µg, 30 µg, and 90 µg of Cefazolin in 10% GelMA. (**B**) Hydrogel degradation in mass loss normalized to initial mass for 5%, 10%, and 15% GelMA and 30 µg of Cefazolin in 5% and 15% GelMA. Data are shown as mean ± standard deviation. The experiment was performed once with n = 4. Cumulative release as the percentage of the respective encapsulation efficiencies from GelMA hydrogels. (**C**) Cumulative release of Cefazolin 3 µg, 15 µg, 30 µg, and 90 µg from 10% GelMA. (**D**) Cumulative release of Cefazolin 30 µg from 5%, 10%, and 15% GelMA. (**E**) Total cefazolin released via enzymatic degradation in µg. Data are shown as mean ± standard deviation. Standard deviations that are not distinguishable are too small thus occluded by the symbols. The experiment was performed once with n = 3.

GelMA hydrogels harbor degradation sites susceptible to metalloproteases such as collagenases and upon implantation undergo proteolytic degradation [39,49]. Thus, the cefazolin release was quantified as the hydrogels were degraded in vitro by collagenase (Figure 3) to simulate in vivo conditions.

In line with our previous work [50], incubation times required to achieve full hydrogel degradation were inversely proportional to the GelMA concentration (Figure 3A,B). Gels with 5% GelMA concentration degraded in 3 h, while 10% GelMA hydrogels degraded within 8 h, and 15% GelMA hydrogels took 13 h to degrade fully. Cefazolin loading with doses ranging from 3–30 µg did not significantly alter degradation profiles of GelMA-DDS based on 10% macromere concentration. However, degradation of 10% GelMA-DDS with 90 µg cefazolin occurred significantly faster than antibiotic-free control groups, suggesting that encapsulation of high cefazolin doses may lead to structural changes in the macromolecular phase, leading to enhanced permeability. In theory, increased hydrogels porosity and permeability can be related to decreased crosslinking density [22,39,51], which would, in turn, improve the access of the enzyme to the hydrogel scaffold, thereby increasing the proteolytic degradation rate [52,53]. This is further supported by the observation of the degradation curve profiles which were approximately linear for higher concentrations of GelMA (10% and 15%), both without loaded cefazolin and with doses ranging between 3 and 30 µg, suggesting a zero-order degradation rate characteristic of surface erosion. Low concentration hydrogels (5% GelMA) and 10% GelMA-DDS with 90 µg cefazolin, on the other hand, displayed more sigmoid-like profiles characteristic of bulk erosion. Together, these observations suggest that hydrogel mesh size, which is inversely proportional to the hydrogel concentration, was reduced by the encapsulation of high cefazolin doses in 10% GelMA, which in turn led to increased enzyme diffusion into the hydrogel favouring bulk erosion of the macromolecular phase [54,55,56,57].

Accelerated proteolytic release of cefazolin presented a burst release in the first two hours. After two hours of release, around 78% to 87% of the drug cargo was released, and complete release was achieved after 4 h for all groups tested (Figure 3C,D). The GelMA concentration did not significantly impact the release kinetics. Yet, similar to the diffusive release, the cefazolin dose encapsulated in the GelMA-DDS dictated the total amount released (Figure 3E). Furthermore, the drug cargo’s full release was achieved before the total degradation of 10% and 15% GelMA hydrogels, indicating that the drug diffused out from the hydrogels faster than degradation occurred.

#### 3.1.4. Mesh Size

The hydrogel mesh size ξ, defined as the distance between consecutive chain crosslinks [58], is a primary characteristic of hydrogel systems and dictates their degradation rate [39], swelling properties [58,59], and the diffusive release of molecules from hydrogel-DDS [15]. Thus, the mesh size is an essential factor in predicting drug release behavior. However, this parameter is difficult to determine experimentally [60] and, consequently, is commonly estimated mathematically based on the equilibrium swelling ratio and the Flory-Rehner equation [24,26,32,58].

**Figure 4:**
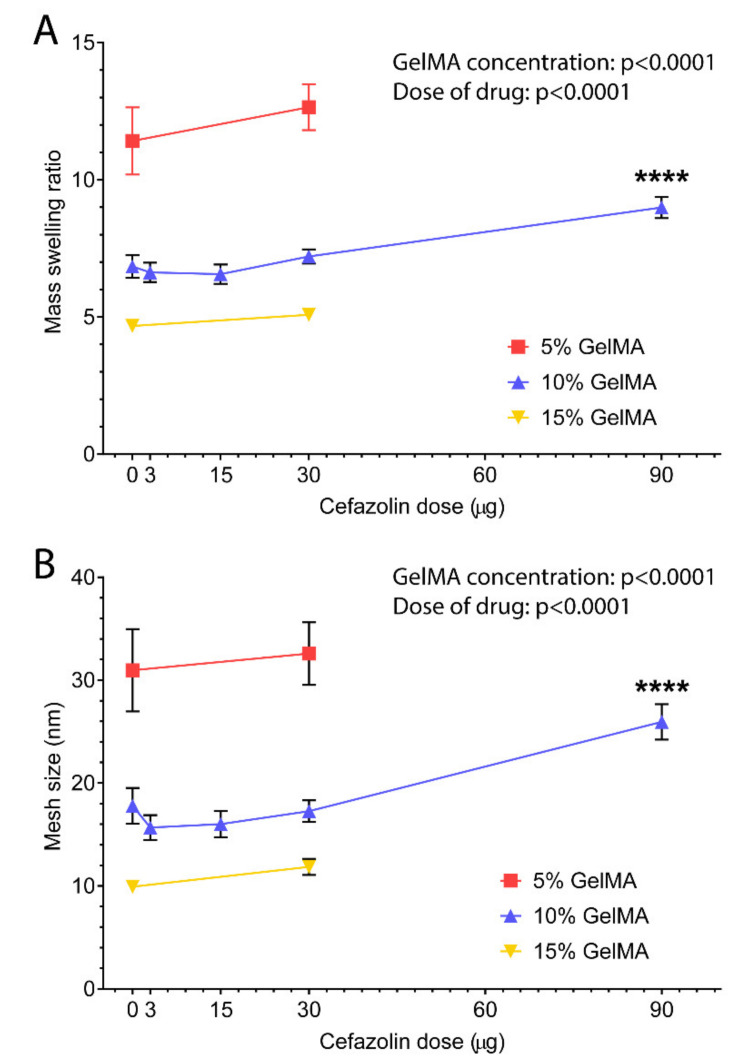
Equilibrium swelling ratio and mesh size of GelMA. GelMA-DDS hydrogels at 5%, 10%, and 15% GelMA for cefazolin doses of 3 µg, 15 µg, 30 µg, and 90 µg. (**A**) Equilibrium swelling ratio after 7 days of swelling in phosphate-saline buffer at 37 °C. (**B**) Mesh size in nm. The experiment was performed once with n = 5–7. **** *p* < 0.0001.

The equilibrium swelling ratio of the GelMA-DDS and the respective control groups presented in Figure 4A displayed a significant effect of the GelMA concentration. The GelMA concentration was inversely correlated with the swelling ratio which decreased from around 11.4 to 4.7 for gels with 5% to 15% macromere concentration. There was also a significant effect of the cefazolin dose loaded in GelMA. The highest dose of cefazolin (90 µg) in 10% GelMA presented an equilibrium swelling ratio of 9, which is significantly higher than the control group (10% GelMA) and closer to the value of the 5% GelMA hydrogels. This may be indicative of a lower crosslinking density that would also translate into a lower hydrogel stiffness.

Similarly, the theoretical hydrogel mesh size decreased with increasing GelMA concentration from 5% to 15% (Figure 4B). The mesh sizes observed ranged from around 10 nm to 31 nm, which is in good agreement with other studies [26]. The dose of drug loading also significantly affected the mesh size in all GelMA concentrations. Most noticeably, the loading of 90 µg cefazolin significantly increased the mesh size of 10% GelMA. Overall, the swelling ratio and calculated mesh size indicate a potential impact of the higher doses of on the physicochemical properties of the GelMA-DDS.

Release profiles are commonly controlled by hydrogel crosslinking density and resulting mesh size [15,50,61]. Here, results showed similar release profiles and burst release for all tested GelMA concentrations, suggesting that mesh sizes, which ranged from 10 nm to 31 nm, did not influence the retention of cefazolin, which has an approximate size of 1.74 nm [45]. Therefore, even at high GelMA concentrations, the smallest mesh size was too large to retain the cefazolin drug loaded in our GelMA-DDS for prolonged periods.

Based on the results of our drug release studies, we concluded that GelMA concentration did not significantly impact the release kinetics, while the cefazolin dose did. The drug was released in a burst release within the first few hours, and the mesh sizes were too large compared to cefazolin to achieve sustained release. However, the objective is to deliver antibiotics locally and the preferred release kinetics is a burst release above the MIC of the bacteria [62]. Indeed, cefazolin release profiles from hydrogel-DDS were reported by others and ranged from a few hours (4–5) to a few days [41,63,64] and are typically characterized by a burst release within the first few hours (1–4) [65,66], followed by an exponential decay [65]. Furthermore, approved DDS for antibiotic release use the dose factor to tailor the release profile, which denotes the primary importance of the dose factor that needs to be above the MIC of the bacteria [67,68]. Additionally, the half-life of cefazolin in the blood compartment is only 1.8–2 h [69,70]. Consequently, our GelMA-DDS provides additional protection and local availability of cefazolin,.

### 3.2. Mechanical and Physical Properties of the Drug Delivery System

#### 3.2.1. Mechanical Testing

When designing an implantable hydrogel-DDS, the mechanical properties of the hydrogel-DDS play a critical role in the system stability and degradability [71]. Therefore, hydrogel mechanical properties should be adaptable to tune degradation and release profiles [71]. We therefore evaluated the tuneability and impact of cefazolin loading on the mechanical properties of GelMA hydrogels. Since the GelMA concentration did not affect the release kinetics of cefazolin, we continued all further experiments with 10% GelMA and cefazolin doses between 3 and 90 µg.

**Figure 5:**
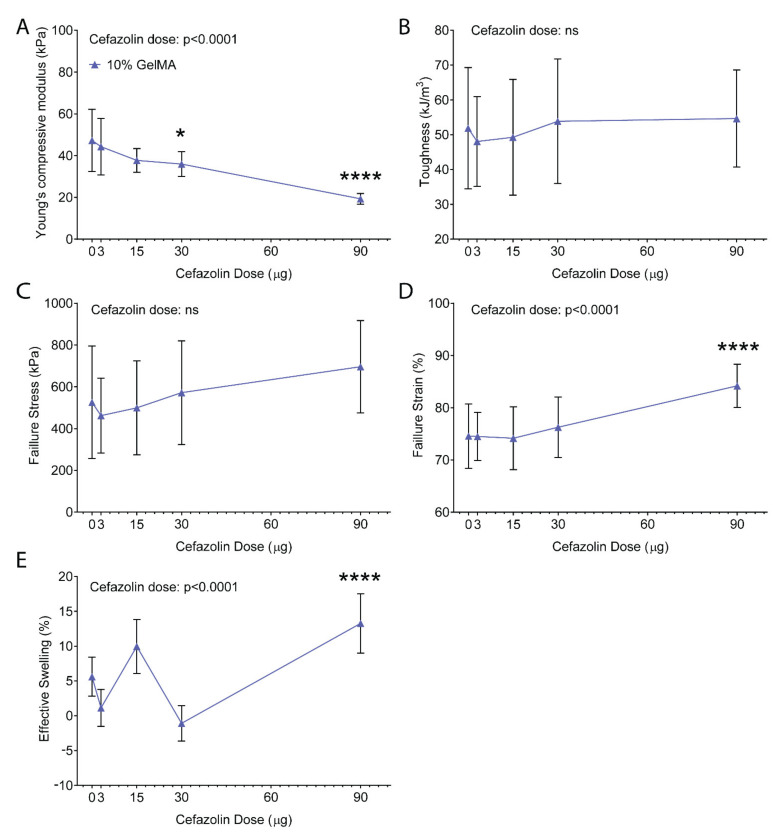
Compressive properties of GelMA-DDS hydrogels at 10% GelMA for cefazolin doses of 0 µg, 3 µg, 15 µg, 30 µg, and 90 µg. (**A**) Compressive modulus. (**B**) Toughness. (**C**) Failure stress. (**D**) Failure strain. (**E**) Effective swelling. Data are shown as mean ± standard deviation, n = 8. * *p* < 0.05; **** *p* < 0.0001. All statistical significances are with reference to the control group (10% GelMA).

Figure 5 shows the mechanical compression testing results for 10% GelMA with and without cefazolin. In the absence of cefazolin, the compressive modulus was ~47 kPa, as expected [72] (Figure 5A). Statistical analysis demonstrated a significant effect of the cefazolin dose on the compressive moduli of the GelMA-DDS (*p* < 0.0001). Indeed, Figure 5A shows that loading of 90 µg cefazolin in 10% GelMA hydrogel discs resulted in a significantly lower compressive modulus than the control group (~19 kPa) which was more similar to the typical modulus of 5% GelMA hydrogels [50,72]. The hydrogel toughness and failure stress averaged from ~48 to ~55 kJ/m^3^ (Figure 5B) and ~462 to ~696 kPa (Figure 5C), respectively, which were unaffected by the cefazolin dose and comparable to previously published data [50,72]. The failure strain was not significantly different between 10% GelMA with and without cefazolin doses ranging from 3 to 30 µg. However, failure strain for the highest cefazolin dose (90 µg) was significantly higher, indicating a lower degree of crosslinking [50,72] (Figure 5D). Effective swelling, a measure of water uptake following incubation in a buffer, ranged from ~−1 to ~10% but was significantly higher at the 90 µg cefazolin dose (~13%), further corroborating lower crosslinking densities in gels containing high doses of cefazolin [50,72] (Figure 5E).

Overall, the expected range of mechanical properties for 10% GelMA observed in our experiment was in accordance with other studies [50,72]. However, encapsulation of the highest doses of cefazolin (90 µg) decreased the compressive moduli, as well as increased the effective swelling and failure strain of GelMA-DDS. The mechanical testing results have thus confirmed and explained the observations that at a fixed GelMA concentration of 10%, higher doses of cefazolin impacted degradation time and mesh size of the final DDS (Figure 3 and Figure 4). Because the mechanical properties of GelMA at a given concentration are dependent on the degree of crosslinking [22], we hypothesized that high concentrations of cefazolin may interfere with the crosslinking reaction. This interference could be related to cefazolin’s molecular structure, which may scavenge free radicals and delay or impair the crosslinking reaction. Evidence of the free-radical scavenging potential of beta-lactam antibiotics, namely ampicillin, penicillin, oxacillin, and dicloxacillin, all belonging to the same antibiotic class as cefazolin, was reported by Berczyński et al. [73].

#### 3.2.2. Photorheology

Our finding that a dose of 90 µg cefazolin decreased the compressive moduli of 10% GelMA-DDS prompted us to investigate the impact of drug loading on the crosslinking reaction. We hypothesized that cefazolin may act as a radical scavenger, and GelMA crosslinking inhibition would increase in a dose-dependent manner. Photocrosslinking of GelMA-DDS was performed via free-radical polymerization using Irgacure 2959, a common photoinitiator for hydrogel systems [74]. Irgacure 2959 is a type I photoinitiator that cleaves into free radical species upon absorption of UV photons [75]. The two free radical species created are a ketyl radicals, which are short-lived, and a benzoyl radicals, which initiate a polymerization chain reaction [75,76]. While the free radical generation is dependent on the light intensity, the crosslinking reaction rate and storage modulus of the hydrogels depends on the concentration of those generated free radicals that initiate the photopolymerization reaction [77].

**Figure 6:**
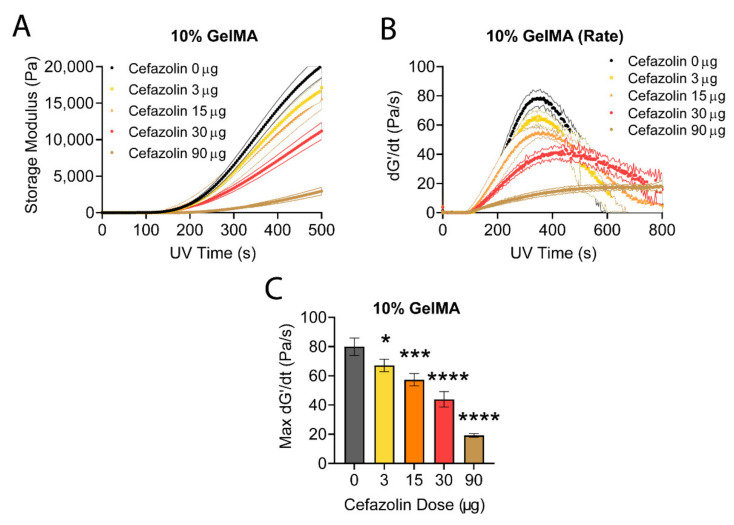
In situ photorheology of 10% GelMA-DDS hydrogels for the first 500 s of the crosslinking reaction. (**A**) Storage modulus. (**B**) Crosslinking reaction rate. (**C**) Maximum crosslinking reaction rates. Photocrosslinking with 0.5 mg/mL Irgacure 2959 at 365 nm. n = 5 samples for all groups. Error bands indicated the standard deviation for A and B. For C, symbols indicate statistical differences compared to the control group without cefazolin (* *p* < 0.05, *** *p* < 0.001, **** *p* < 0.0001).

The photocrosslinking reaction was empirically studied in real-time by monitoring the storage modulus (*G’*) as a function of UV exposure time (Figure 6A) for 3 to 90 µg cefazolin in 10% GelMA. We observed a delay (onset time) after the UV light was turned on before an increase in *G’* was detected. This onset time (~150 s) is attributed to the presence of oxygen in the hydrogel precursor solution, which scavenges the photoinitiator radicals [77]. This dissolved oxygen must be depleted before the crosslinking reaction is underway. The storage moduli (Figure 6A) and photocrosslinking reaction rates (Figure 6B) were inversely correlated with the encapsulated cefazolin dose. The maximum crosslinking rates, shown in Figure 6C, confirmed the dose-dependent effect of cefazolin on the crosslinking reaction rate, which decreased as a function of the increasing cefazolin dose. Collectively, this data demonstrates that the presence of high doses/concentrations of cefazolin impacted the polymerization of the GelMA-DDS.

There may be different explanations for this observation. In free-radical polymerizations, the rate of propagation (*Rp*) is proportional to the square root of the rate of initiation (*Ri*) [78], according to:

(7) $$R_{p}=k_{p}\left[M\right]{\left(\frac{R_{i}}{2k_{t}}\right)}^{\frac{1}{2}}$$

Here [*M*] is the concentration of monomer, *k_p_* is the rate constant for propagation, and *k_t_* is the rate constant for termination. The rate of initiation is given by [78]:

(8) $$R_{i}=\frac{2\varphi \varepsilon f\lambda Ic_{i}}{N_{A}hc}$$

where *φ* is the quantum yield of photolysis i.e., the fraction of absorbed photons that cleave a photoinitiator molecule (0.29); *ε* is the molar extinction coefficient (2.46 M^−1^ cm^−1^ [77]); *f* is the photoinitiator efficiency (the ratio of initiation events to radicals generated by photolysis); *λ* is the wavelength of light (365 nm); *I* is the intensity of light (0.003 W/cm^2^ [77]); *c_i_* is the instantaneous molar concentration of photoinitiator molecules (2.23 × 10^−3^ M); *N_A_* is Avogadro’s constant (6.022 × 10^23^ mol^−1^); *h* is Planck’s constant (6.63 × 10^−34^ m^2^ kg/s); *c* is the speed of light (299,792,458 m/s).

There are three factors from the two previously mentioned equations (Equations (7) and (8)) that may be affected by the loading of cefazolin in GelMA: the light intensity *I*, the photoinitiator efficiency *f*, and the rate of termination *k_t_*. Firstly, the light intensity *I*; this is unlikely to be affected by the cefazolin loading since the UV-Vis spectrum of cefazolin shows negligible absorbance at 365 nm [79]. Secondly, the photoinitiator efficiency *f*; if free radicals reacted with the drug, this would reduce the proportion available to initiate crosslinking. In that case, the reaction rate should show a square root dependence on the cefazolin concentration. Thirdly, if the radicalized GelMA molecules reacted with the drug, this might increase the rate of termination *k_t_*. This would reduce the rate of crosslinking, may also reduce the final storage modulus achieved, and would also display a crosslinking rate dependent on the square root dependence on the cefazolin concentration. Considering the relative concentrations and mobilities of each species, we suspect the photoinitiator efficiency is the parameter most likely affected by cefazolin loading.

**Figure 7:**
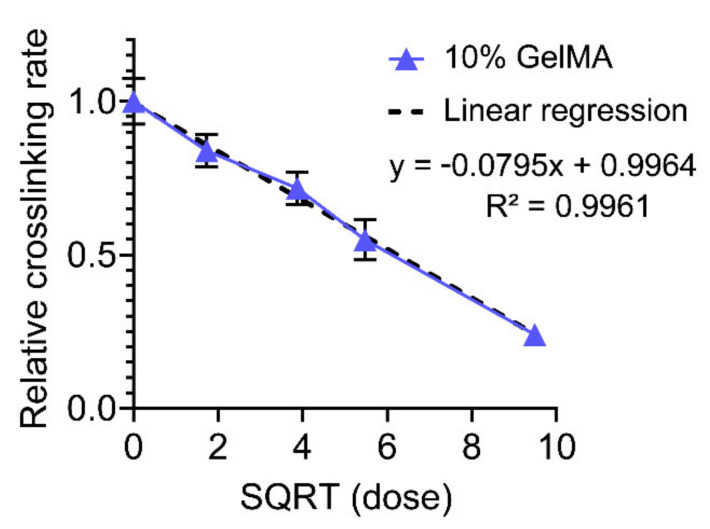
10% GelMA crosslinking rate normalized to the control sample (no cefazolin) versus the square roots of cefazolin doses. Linear regression fit showing good agreement: R^2^ = 0.9961.

To investigate our hypothesis, the crosslinking rates were normalized to the control group, 10% GelMA containing no cefazolin, and plotted against the square root of the cefazolin doses (Figure 7). The reaction rates show a linear dependence with the square root of the cefazolin concentration, as it would be expected if cefazolin is reacting with some of the initializing free radicals. The linear regression fit in Figure 7 confirmed the relationship with an *R^2^* superior to 0.99.

Once we confirmed the impact of the cefazolin loading on the crosslinking rate and the photoinitiator efficiency *f* (proportional to the square root of the crosslinking rate), we then mathematically estimated the proportion of cefazolin molecules consumed by free radicals [77] (Table 2).

The in situ photorheology characterization indicated a significant and robust relationship between cefazolin concentration and the rate of the photocrosslinking reaction. While the radicalized initiators may be reacting with cefazolin, reducing the effective concentration of initiating free radicals, the effect on the cefazolin loading was limited to a maximum of 3% of the cefazolin that would be consumed by free radicals. This result implies that a maximum of ~90 ng out of the 3 µg of cefazolin would be consumed, while ~480 ng from the 90 µg of cefazolin would be consumed.

Furthermore, while all cefazolin doses impacted the crosslinking reaction rate in a dose-dependent manner, only the highest dose of 90 µg of cefazolin significantly decreased the final physicochemical properties of the GelMA-DDS in the fully crosslinked hydrogels (Figure 5).

### 3.3. In Vitro Evaluation of GelMA-DDS

#### 3.3.1. Diffusive Zone of Inhibition Assay

**Figure 8:**
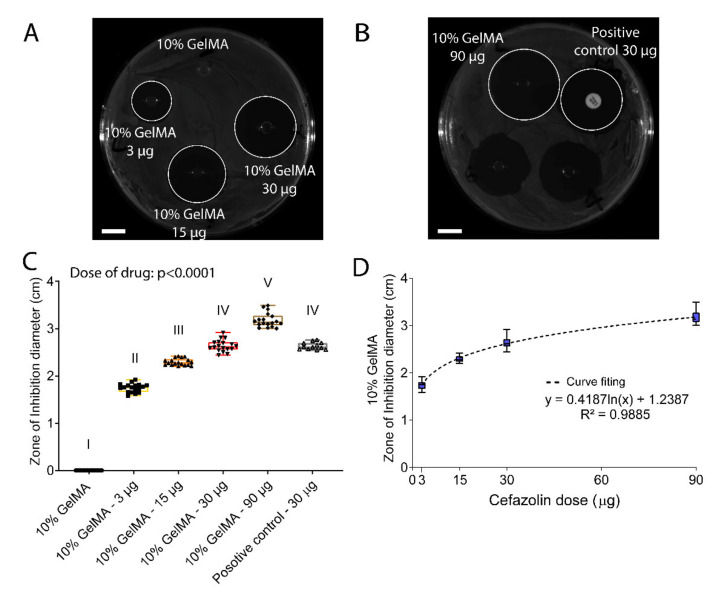
In vitro evaluation of 10% GelMA-DDS efficacy against *S. aureus* in a zone of inhibition assay. *S. aureus* was spread on Petri dishes containing Mueller-Hinton agar, then samples were placed on the culture plate and left incubating at 37 °C overnight. (**A**,**B**) Representative pictures of Petrie dishes at the end of the assay. Scale bar = 1 cm. (**C**) Zone of inhibition in cm. Results are shown as box plots. Groups with no statistical difference are marked with the same roman numeral. (**D**) Non-linear regression for GelMA 10% versus cefazolin doses. The experiment was performed three times with n = 6 each time.

The efficacy of the prophylactic release of cefazolin from GelMA was first assessed against *S. aureus,* one of the main pathogens involved in the SSI incidence [3], using a zone inhibition assay (Figure 8). Representative images of the zone of inhibition for all tested groups are presented in Figure 8A,B. As expected, the zone of inhibition increased in a dose-dependent manner from 3 µg, 15 µg, 30 µg, and 90 µg released from 10% GelMA hydrogels following a logarithmic function (Figure 8C,D). A similar dose-dependency effect of cefazolin was observed when the antibiotic was released from polycaprolactone scaffolds in another study [80]. Additionally, the 30-µg cefazolin dose had a similar efficiency compared to the positive control with the same dose loaded; thus, the cefazolin bioactivity was not altered despite small amounts of cefazolin being consumed during the photocrosslinking reaction (Table 2).

#### 3.3.2. Broth Growth Inhibition Assay

**Figure 9:**
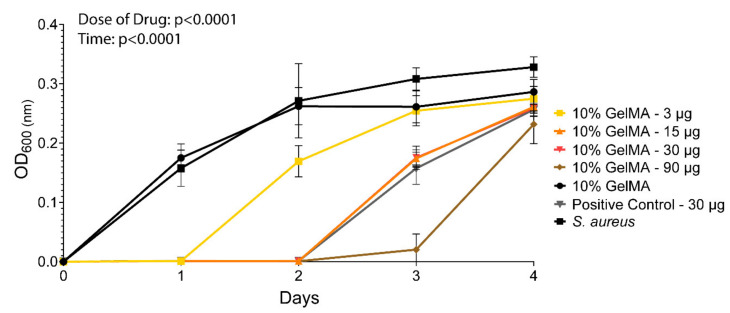
In vitro evaluation of 10% GelMA-DDS efficacy against broth culture of *S. aureus***.** A 0.5 McFarland of *S. aureus* suspension in MH broth was prepared and diluted 100 times to reach 5 × 10^6^ CFU/mL. The broth suspension was added to each well to start the assay (0.5 mL per well), and a new broth solution was prepared every day, at every timepoint except for the last one, to refresh the bacteria solution. Optical density was measured at 600 nm each day for all groups: 3 µg, 15 µg, 30 µg, and 90 µg cefazolin in 10% GelMA. Control groups were added: 10% GelMA = negative control; *S. aureus* = negative control; positive control disc containing cefazolin 30 µg. Data are shown as mean ± SD. The experiment was performed three times with n = 6.

The zone of inhibitions assay was complemented by a broth inhibition assay designed based on the microdilution susceptibility assay [81]. The goal was to assess the antibacterial efficacy of cefazolin loaded GelMA-DDS against *S. aureus* in suspension and over time [40,82]. Figure 9 shows that the dose was a significant factor affecting *S. aureus* growth inhibition. A cefazolin dose of 3 µg inhibited bacteria growth for 1 day only, while the doses 15 µg and 30 µg both inhibited bacteria growth for 2 days, and 90 µg of cefazolin prevented bacteria growth for 3 days. Additionally, the cefazolin dose of 30 µg showed similar antibacterial efficacy compared to the positive control of cefazolin 30 µg on day 0. In this assay, we saw a prolonged antibacterial effect of cefazolin for up to several days, while the diffusive release assay performed earlier showed that most of the drug cargo was released within hours (Figure 2). The MIC of cefazolin for *S. aureus* ATCC29213 inoculated at 5 × 10^6^ CFU/mL is close to 1 µg/mL [37]. Assuming that the cefazolin concentration in GelMA and the media were in equilibrium, a fraction of the cefazolin dose would remain in GelMA after day 1. These fractions are equivalent to 6.3 µg, 2.1 µg, 1.05 µg, and 0.21 µg, for total drug doses of 90 µg, 30 µg, 15 µg, and 3 µg, respectively. Therefore, cefazolin amounts remaining in the hydrogel-DDS on day 2 were all above the MIC except for the lowest dose of 3 µg cefazolin, resulting in the observed antibacterial effects. Additionally, contrary to the diffusive release in PBS (Figure 2), where the release media was refreshed four times during the first 24 h which would induce an overall higher osmotic pressure that favored the drug release, the MH broth was refreshed only once a day in the bacteria broth assay, thereby reducing the diffusion rate of the drug in the media [83].

## 4. Summary and Conclusions

The developed GelMA-DDS allows for high drug loading efficiencies of cefazolin from 65% to 99%. Although cefazolin impacted the crosslinking reaction rate in a dose-dependent manner, the mechanical properties of the GelMA-DDS were overall unaffected up to the 30-µg dose. In vitro release characterization revealed a burst release of cefazolin within four hours for all experimental groups in both the diffusive and the proteolytic release. While the GelMA concentrations did not impact the release kinetics, the cefazolin dose enabled the tailoring of the absolute drug doses released, which may be useful in preventing SSI and implant-related infections. The swelling ratio and hydrogel mesh size were successfully tailored, excluding the 90-µg cefazolin dose, which impacted those characteristics due to cefazolin’s scavenging of free radicals during the polymerization reaction. Therefore, antibiotic loading up to 30 µg are recommended. Within the range tested, all hydrogel mesh sizes were larger than the cefazolin molecules, suggesting that the drug diffused out of the hydrogel with ease. Finally, the cefazolin-loaded GelMA-DDS retained its in vitro antibiotic activity and inhibited the growth of *S. aureus* in a dose-dependent manner.

This study focused on the in vitro mechanisms of the drug release and the hydrogel-DDS characterization, all necessary for the GelMA-DDS development. The next step required is the definition of appropriate doses for an in vivo infection model where the dose design would depend primarily on the infection inoculum, the size of the defect, and the potential negative impact of the drug on the DDS mechanical properties [84,85,86]. The GelMA-DDS provides a simple and elegant solution to SSI prophylaxis and may reduce systemic side-effects associated with traditional treatments by providing a local administration of the drug-loaded tailorable hydrogel.

## Figures and Tables

**Table 1:** Experimental groups denomination and composition.

Group Denomination	GelMA Concentration (%)	Cefazolin Dose (µg)
5% GelMA	5	0
5% GelMA–30 µg	5	30
10% GelMA	10	0
10% GelMA–3 µg	10	3
10% GelMA–15 µg	10	15
10% GelMA–30 µg	10	30
10% GelMA–90 µg	10	90
15% GelMA	15	0
15% GelMA–30 µg	15	30

**Table 2:** Proportion of cefazolin molecules potentially consumed during crosslinking reaction.

Cefazolin Dose (µg)	Cefazolin Concentration (M)	Relative Crosslinking Rate (%)	Photoinitiator Efficiency f:	Effective Rate of Free Radical Generation (M/s)	Cumulative Consumed Radicals (M) at t = 1800s	Proportion of Drug Consumed	Mass of Drug Consumed (ng)
**0**	0	1.00	1	3.33 × 10^−8^	0.0	0.00%	0
**3**	1.89 × 10^−4^	0.81	0.9	3.00 × 10^−8^	5.9 × 10^−6^	3.14%	94.20
**15**	9.43 × 10^−4^	0.69	0.8307	2.77 × 10^−8^	1.0 × 10^−5^	1.07%	160.50
**30**	1.89 × 10^−3^	0.59	0.7681	2.56 × 10^−8^	1.4 × 10^−5^	0.72%	216.00
**90**	5.66 × 10^−3^	0.24	0.4899	1.63 × 10^−8^	3.0 × 10^−5^	0.53%	477.00

## Data Availability

The data presented in this study are available on request from the corresponding authors.

## Supplementary Materials

The following are available online at www.mdpi.com/2073-4360/13/22/3960/s1, Figure S1. High-performance liquid chromatography coupled with mass spectrometry detection of Cefazolin. Comparison of standard curves for Cefazolin detected in a Phosphate-buffered saline (PBS) solution and in a collagenase II and Hank’s Balanced Salt Solution (HBSS). The standard curves covered the range of 1.95 ppb = ng/mL to 1000 ppb = 1000 ng/mL. (A) Standard curve. (B) Column comparison.; Figure S2. Cefazolin diffusive in vitro release in phosphate-buffered saline at pH 7.4, under 75 rpm agitation at 37 °C. 3 µg, 15 µg, 30 µg, and 90 µg cefazolin were released from 5%, 10%, and 15% GelMA. (A) Cefazolin total release in µg. (B) Fraction of cefazolin released per timepoint expressed in µg.; Figure S3. Gelatin methacryloyl hydrogel at 5%, 10%, and 15% concentration loaded with cefazolin 3 µg, 15 µg, 30 µg, and 90 µg were swelled in in phosphate-buffered saline at pH 7.4 for 7 days to ensure reaching the equilibrium swelling. (A) Weight of the hydrogels at equilibrium swelling. (B) Weight of the hydrogels after lyophylization. N = 5–7.; Figure S4. Compressive properties of GelMA-DDS hydrogels at 5%, 10%, and 15% GelMA for cefazolin doses of 0 µg, 3 µg, 15 µg, 30 µg, and 90 µg. (A) Compressive modulus. (B) Toughness. (C) Failure stress. (D) Failure strain. (E) Effective swelling. Data are shown as mean ± standard deviation, n = 8. The mention ‘GelMA concentration X Cefazolin dose’ signifies that the interaction of both parameters is significant. * *p* < 0.05; *** *p* < 0.001, **** *p* < 0.0001. All statistical significances are with reference to the respective control groups: 5% GelMA, 10% GelMA, 15% GelMA. # indicates a significant difference between 5% GelMA + Cefazolin 30 μg and 5% GelMA.; Figure S5. In situ photorheology for 5%, 10%, and 15% GelMA containing cefazolin with 0.5% Irgacure2959 photoinitiator. (A) Raw data for 5% GelMA, 5% GelMA + 30 µg of cefazolin, and 5% GelMA + 90 µg of cefazolin. (B) Raw data for 10% GelMA, 10% GelMA + 3 of µg cefazolin, 10% GelMA + 15 of µg cefazolin, 10% GelMA + 30 of µg cefazolin, and 10% GelMA + 90 of µg cefazolin. (C) Raw data for 15% GelMA, 15% GelMA + 30 µg of cefazolin, and 15% GelMA + 90 µg of cefazolin.; Figure S6. In situ photorheology of GelMA-DDS hydrogels for the first 500 s of the crosslinking reaction. (A) 5% GelMA storage modulus. (B) 5% GelMA crosslinking reaction rate. (C) 15% GelMA storage modulus. (D) 15% GelMA crosslinking reaction rate. (E) 5% GelMA maximum crosslinking reaction rates. (F) 15% GelMA maximum crosslinking reaction rates. Photocrosslinking with 0.5 mg/mL Irgacure 2959 at 365 nm. n = 5 for all 5% GelMA and n = 2 for all 15% GelMA samples. Error bands indicated the standard deviation for A to D. For E, symbols indicate statistical differences compared to control groups without cefazolin (* *p* < 0.05; ** *p* < 0.01, *** *p* < 0.001, **** *p* < 0.0001).; Figure S7. Normalized crosslinking rates against the control sample (no cefazolin) at 5% and 15% GelMA. (A) Relative crosslinking rate versus the square roots of cefazolin doses. (B) Linear regression fit showing good agreement: R2 = 0.995.; Figure S8. Free radicals used for the crosslinking reaction. (A) Photoinitiator degradation model in a 1 cm cube. Incident light intensity (in einsteins s^−1^ cm^−2^) × fraction of light absorbed × quantum yield = degradation rate of photo-initiator (in moles s^−1^). Modified from [1]. (B) Estimate of I2959 radicals generated as a function of time for in absence of cefazolin 0 µg and for the different cefazolin doses, 3 µg, 15 µg, 30 µg, and 90 µg, using the respective photoinitiator efficiencies.; Figure S9. In vitro evaluation of 5% and 15% GelMA-DDS efficacy against *S. aureus* in a zone of inhibition assay. *S. aureus* was spread on Petri dishes containing Mueller-Hinton agar, then samples were placed on the culture plate and left incubating at 37 °C overnight. (A) and (B) Representative pictures of Petrie dishes at the end of the assay. Scale bar = 1 cm. (C) Zone of inhibition in cm. Results are shown as box plots. Groups with no statistical difference are marked with the same roman numeral. The experiment was performed three times with n = 6 each time.; Figure S10. In vitro evaluation of 10% GelMA-DDS efficacy against broth culture of *S. aureus*. A 0.5 McFarland of *S. aureus* suspension in MH broth was prepared and diluted 100 times to reach 5 × 106 CFU/mL. The broth suspension was added to each well to start the assay (0.5 mL per well), and a new broth solution was prepared every day, at every timepoint except for the last one, to refresh the bacteria solution. Optical density was measured at 600 nm each day for all groups: 30 µg cefazolin in 5%, 10%, and 15% GelMA. Control groups were added: 10% GelMA = negative control; *S. aureus* = negative control; positive control disc containing cefazolin 30 µg. Data are shown as mean ± SD. The experiment was performed three times with n = 6.
