# Supplementary material for: Gelatin Methacryloyl Hydrogels for the Localized Delivery of Cefazolin
Source: Polymers (Basel). 2021 Nov 16;13(22):3960. doi: 10.3390/polym13223960 (PMC8618379; doi:10.3390/polym13223960)
Supplement: Supplementary file 1 [file polymers-13-03960-s001.zip › polymers-1460224-supplementary.pdf]

SUPPLEMENTARY MATERIAL: GELATIN METHACRYLOYL HYDROGELS FOR THE LOCALIZED DELIVERY OF CEFAZOLIN

Margaux Vigata [1], Cathal D. O'Connell [2,3], Silvia C. Cometta Conde [1], Dietmar W. Hutmacher [1,4,5], Christoph Meinert [1,6,] and Nathalie Bock [4,7,8,*]*

[1] School of Mechanical, Medical and Process Engineering, Science and Engineering Faculty (SEF), QUT, Brisbane, QLD, Australia.

[2] BioFab3D-ACMD-St Vincent's Hospital Melbourne, Australia

[3] Discipline of Electrical and Biomedical Engineering, School of Engineering, RMIT University, Melbourne, Australia

[4] School of Biomedical Sciences, Faculty of Health, QUT, Brisbane, QLD, Australia.

[5] Australian Research Council Industrial Transformation Training Centre in Additive Biomanufacturing, QUT, Brisbane, Queensland 4059, Australia.

[6] Herston Biofabrication Institute, Metro North Hospital and Health Services, Brisbane, QLD, Australia

[7] Translational Research Institute, Woolloongabba QLD 4102, Australia.

[8] ARC Training Centre for Multiscale 3D Imaging, Modelling and Manufacturing (M3D Innovation), QUT, Kelvin Grove, 4059, QLD, Australia

* Correspondence: NB: n.bock@qut.edu.au; Tel.: +61 (0)7 3443 7343; CM: christoph.meinert@qut.edu.au; Tel.: +61 (0) 450 177 179.

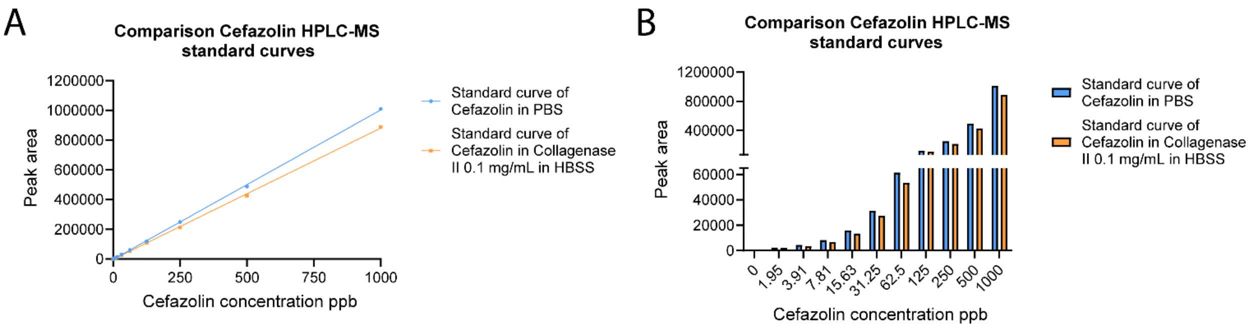

Figure S1. *High-performance liquid chromatography coupled with mass spectrometry detection of Cefazolin. Comparison of standard curves for Cefazolin detected in a Phosphate-buffered saline (PBS) solution and in a collagenase II and Hank's Balanced Salt Solution (HBSS). The standard curves covered the range of 1.95 ppb = ng/mL to 1000 ppb = 1000 ng/mL. A) Standard curve. B) Column comparison.*

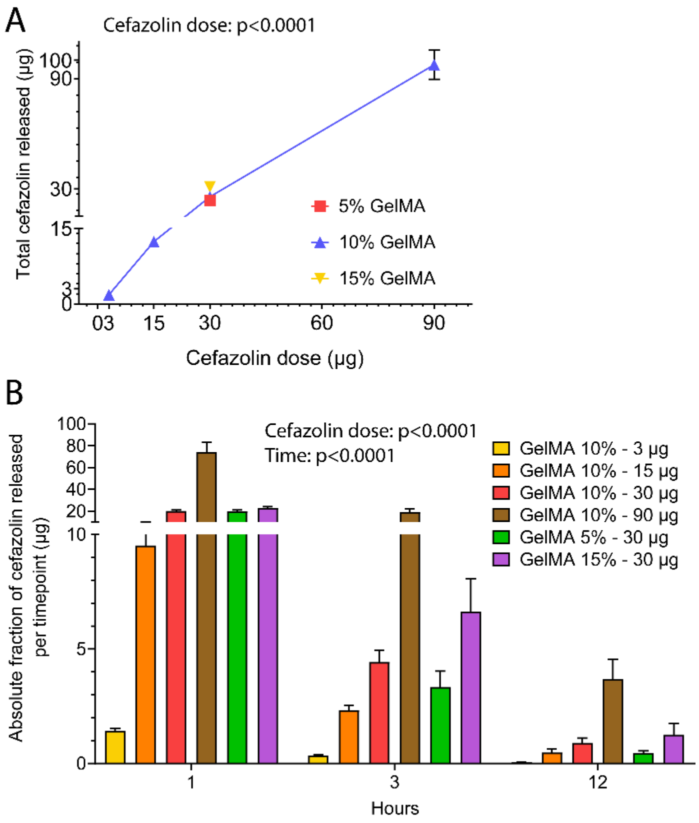

Figure S2. *Cefazolin diffusive in vitro release in phosphate-buffered saline at pH 7.4, under 75 rpm agitation at 37 °C. 3 µg, 15 µg, 30 µg, and 90 µg cefazolin were released from 5%, 10%, and 15% GelMA. A) Cefazolin total release in µg. B) Fraction of cefazolin released per timepoint expressed in µg.*

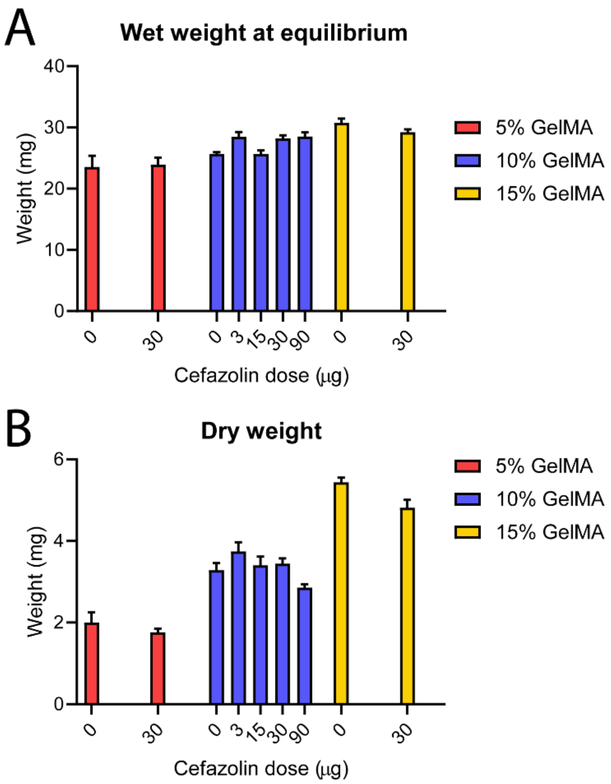

Figure S3. *Gelatin methacryloyl hydrogel at 5%, 10%, and 15% concentration loaded with cefazolin 3 µg, 15 µg, 30 µg, and 90 µg were swelled in in phosphate-buffered saline at pH 7.4 for 7 days to ensure reaching the equilibrium swelling. A) Weight of the hydrogels at equilibrium swelling. B) Weight of the hydrogels after lyophylization. N = 5-7.*

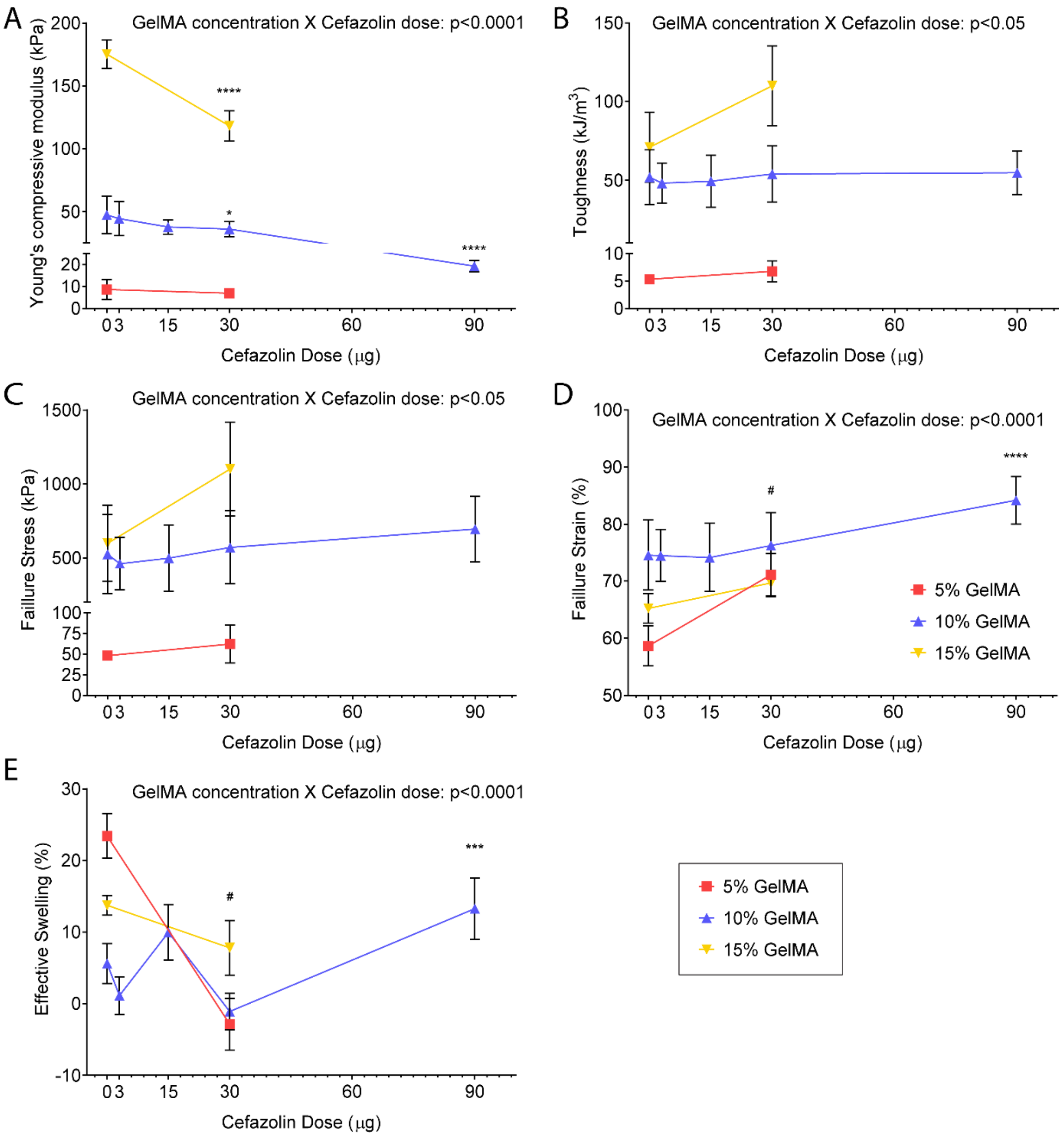

Figure S4. *Compressive properties of GelMA-DDS hydrogels at 5%, 10%, and 15% GelMA for cefazolin doses of 0 µg, 3 µg, 15 µg, 30 µg, and 90 µg. A) Compressive modulus. B) Toughness. C) Failure stress. D) Failure strain. E) Effective swelling. Data are shown as mean ± standard deviation, n = 8. The mention 'GelMA concentration X Cefazolin dose' signifies that the interaction of both parameters is significant. * p < 0.05; *** p<0.001, **** p < 0.0001. All statistical significances are with reference to the respective control groups: 5% GelMA, 10% GelMA, 15% GelMA. # indicates a significant difference between 5% GelMA + Cefazolin 30 µg and 5% GelMA.*

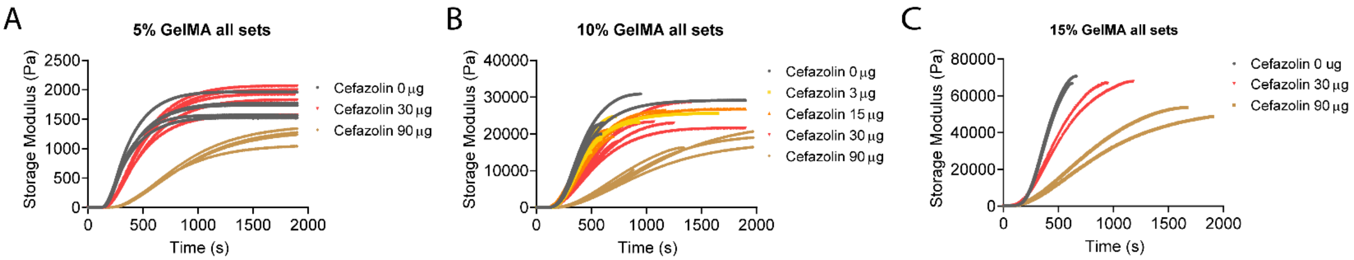

Figure S5. *In situ photorheology for 5%, 10%, and 15% GelMA containing cefazolin with 0.5% Irgacure2959 photoinitiator. A) Raw data for 5% GelMA, 5% GelMA + 30 μg of cefazolin, and 5% GelMA + 90 μg of cefazolin. B) Raw data for 10% GelMA, 10% GelMA + 3 of μg cefazolin, 10% GelMA + 15 of μg cefazolin, 10% GelMA + 30 of μg cefazolin, and 10% GelMA + 90 of μg cefazolin. C) Raw data for 15% GelMA, 15% GelMA + 30 μg of cefazolin, and 15% GelMA + 90 μg of cefazolin.*

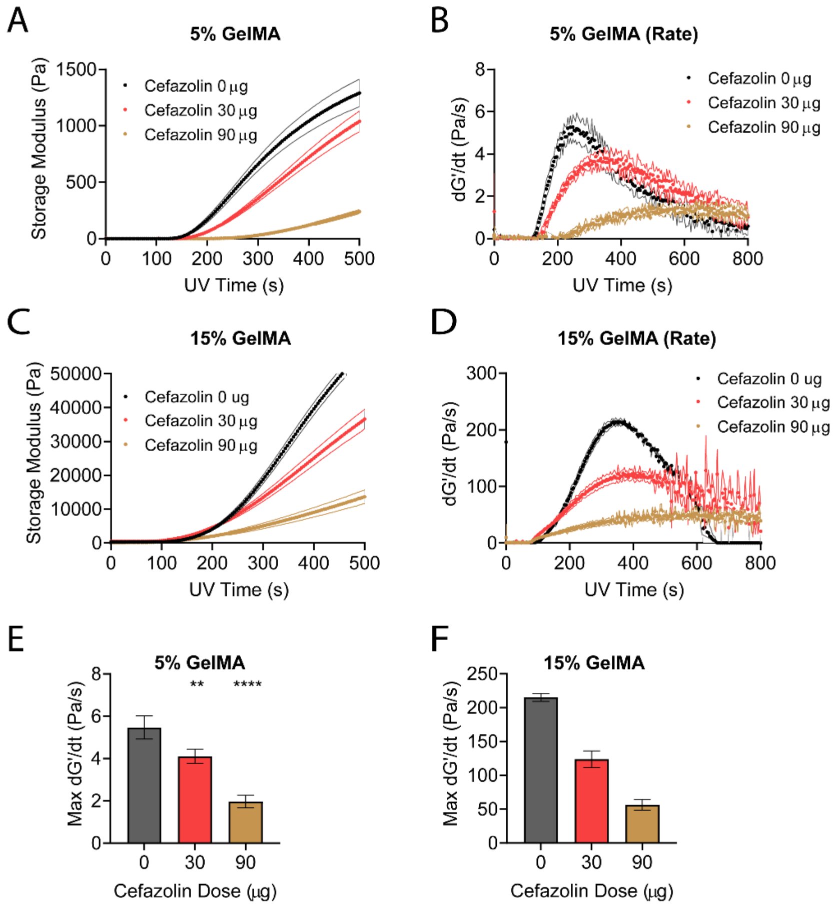

Figure S6. *In situ photorheology of GelMA-DDS hydrogels for the first 500 seconds of the crosslinking reaction. A) 5% GelMA storage modulus. B) 5% GelMA crosslinking reaction rate. C) 15% GelMA storage modulus. D) 15% GelMA crosslinking reaction rate. E) 5% GelMA maximum crosslinking reaction rates. F) 15% GelMA maximum crosslinking reaction rates. Photocrosslinking with 0.5 mg/mL Irgacure 2959 at 365 nm. n=5 for all 5% GelMA and n=2 for all 15% GelMA samples. Error bands indicated the standard deviation for A to D. For E, symbols indicate statistical differences compared to control groups without cefazolin (* $p < 0.05$; ** $P < 0.01$, *** $p<0.001$, **** $p < 0.0001$).*

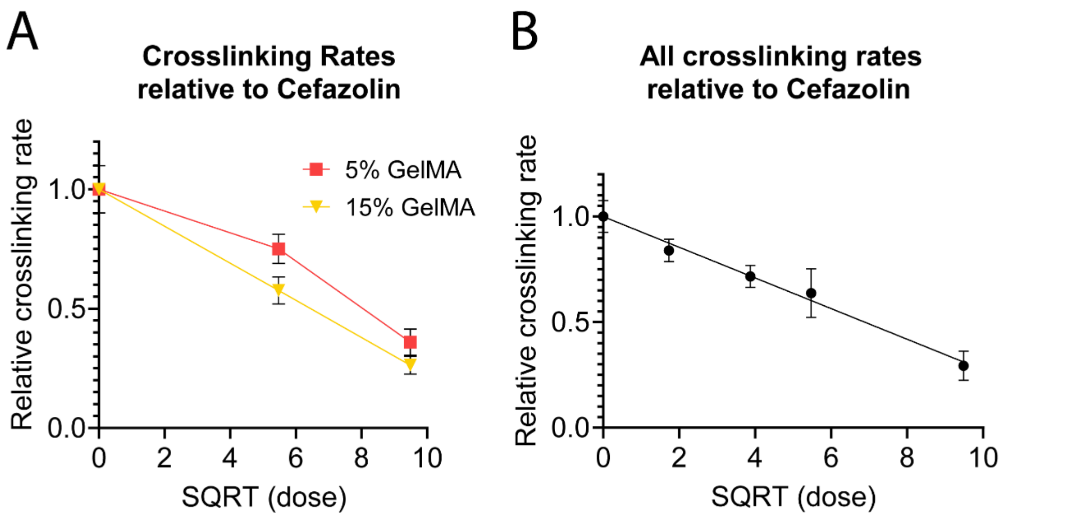

Figure S7. *Normalized crosslinking rates against the control sample (no cefazolin) at 5% and 15% GelMA. A) Relative crosslinking rate versus the square roots of cefazolin doses. B) Linear regression fit showing good agreement: R2 = 0.995.*

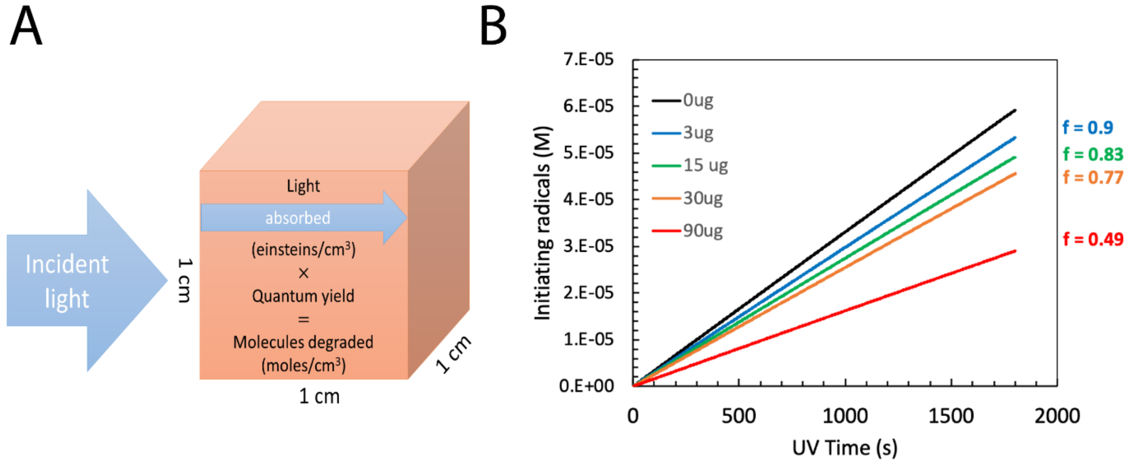

Figure S8. *Free radicals used for the crosslinking reaction. A) Photoinitiator degradation model in a 1 cm cube. Incident light intensity (in einsteins s-1 cm-2) x fraction of light absorbed x quantum yield = degradation rate of photo-initiator (in moles s-1). Modified from [1]. B) Estimate of I2959 radicals generated as a function of time for in absence of cefazolin 0 µg and for the different cefazolin doses, 3 µg, 15 µg, 30 µg, and 90 µg, using the respective photoinitiator efficiencies.*

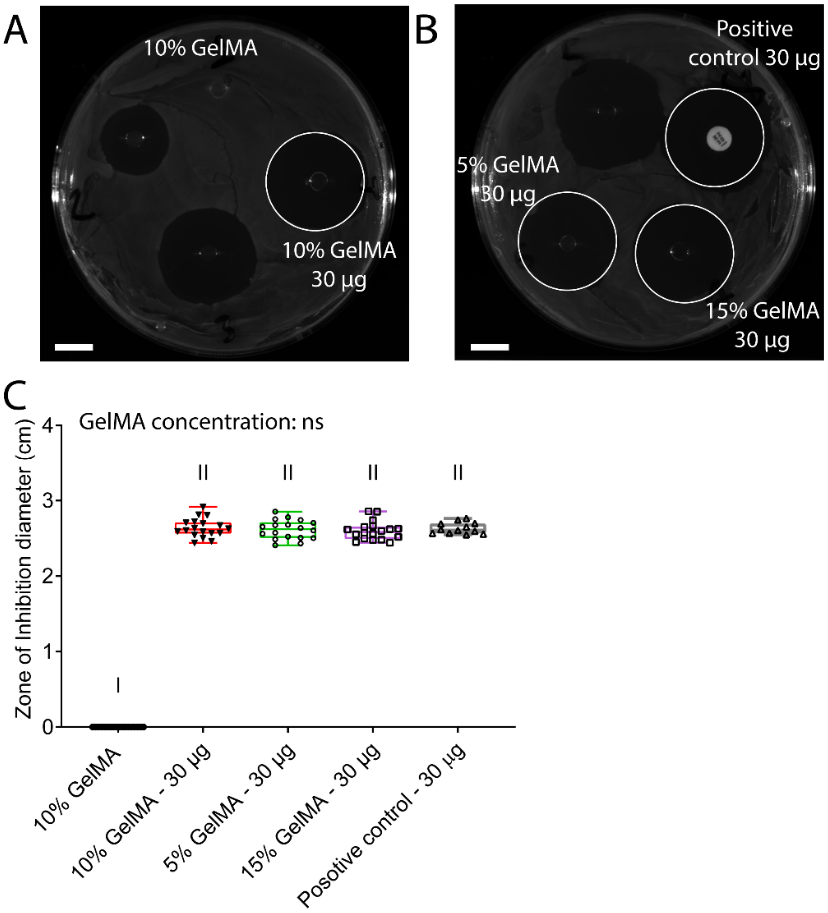

Figure S9. *In vitro evaluation of 5% and 15% GelMA-DDS efficacy against S .aureus in a zone of inhibition assay. S. aureus was spread on Petri dishes containing Mueller-Hinton agar, then samples were placed on the culture plate and left incubating at 37°C overnight. A) and B) Representative pictures of Petrie dishes at the end of the assay. Scale bar = 1 cm. C) Zone of inhibition in cm. Results are shown as box plots. Groups with no statistical difference are marked with the same roman numeral. The experiment was performed three times with n = 6 each time.*

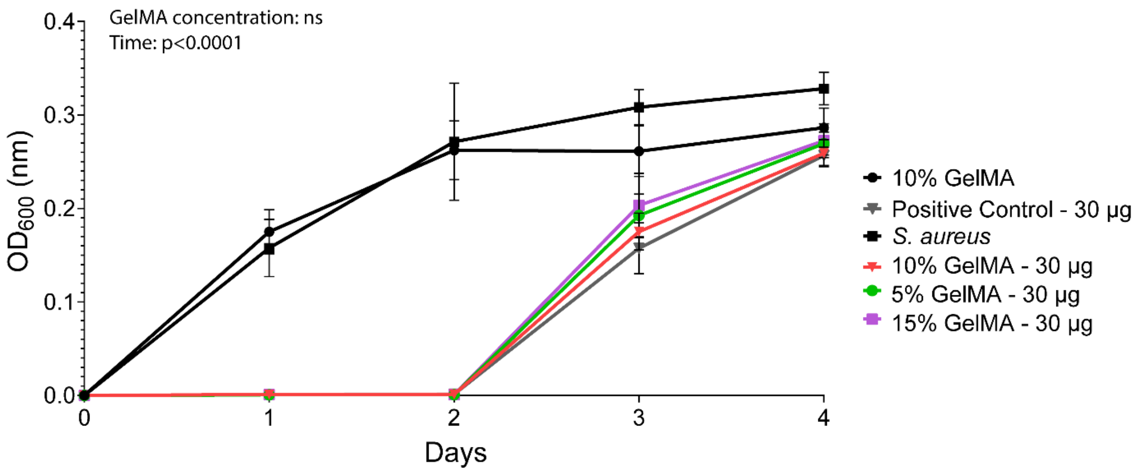

Figure S10. *In vitro evaluation of 10% GelMA-DDS efficacy against broth culture of S.aureus. A 0.5 McFarland of S. aureus suspension in MH broth was prepared and diluted 100 times to reach 5 × 106 CFU/mL. The broth suspension was added to each well to start the assay (0.5 mL per well), and a new broth solution was prepared every day, at every timepoint except for the last one, to refresh the bacteria solution. Optical density was measured at 600 nm each day for all groups: 30 µg cefazolin in 5%, 10%, and 15% GelMA. Control groups were added: 10% GelMA = negative control; S. aureus= negative control; positive control disc containing cefazolin 30 µg. Data are shown as mean ± SD. The experiment was performed three times with n = 6.*

References

[1] O'Connell, C. D. et al. Tailoring the mechanical properties of gelatin methacryloyl hydrogels through manipulation of the photocrosslinking conditions. Soft Matter 14, 2142–2151 (2018).
